# Changes in incidence and etiology of early-onset neonatal infections 1997–2017 – a retrospective cohort study in western Sweden

**DOI:** 10.1186/s12887-019-1866-z

**Published:** 2019-12-12

**Authors:** Margrét Johansson Gudjónsdóttir, Anders Elfvin, Elisabet Hentz, Ingegerd Adlerberth, Ingemar Tessin, Birger Trollfors

**Affiliations:** 1 Department of Pediatrics, Institute of Clinical Sciences, Sahlgrenska Academy, University of Gothenburg, Gothenburg, Sweden; 2 Region Västra Götaland, Sahlgrenska University Hospital, Department of Pediatrics, Gothenburg, Sweden; 3 Department of infectious Diseases, Institute of Biomedicine, Sahlgrenska Academy, University of Gothenburg, Gothenburg, Sweden

**Keywords:** NEONATAL SEPSIS, MENINGITIS, EARLY ONSET, EPIDEMIOLOGY

## Abstract

**Background:**

The objective of the study was to evaluate data on early-onset neonatal invasive infections in western Sweden for the period 1997–2017. To identify changes in incidence, etiology and mortality and compare to previous studies from the same area starting from 1975.

**Methods:**

Observational epidemiological, retrospective study on infants 0–6 days of age with a positive culture in blood and/or cerebrospinal fluid between 1997 and 2017. A comparison was made of the incidence between 2008 and 2017 compared to 1997–2007. Changes in the incidence of infections due to Group B streptococci, *Staphylococcus aureus* and aerobic Gram-negative rods were assessed from 1975.

**Results:**

The total incidence, including both recognized pathogens and commensals as causative agents, was 1.1/1000 live births. The incidence declined from 1.4/1000 LB in 1997–2007 to 0.9/1000 LB in 2008–2017 but the case-fatality rate remained unchanged, (8/119 vs 7/90), at 7%. Among the 209 patients identified during 1997–2017 with sepsis or meningitis the most common organisms were Group B streptococci (40%, 84/209), *S. aureus* (16%, 33/209) and *E. coli* (9%, 18/209). The incidence of Group B streptococci infections went from 0.9/1000 live births 1987–1996 to 0.45/1000 live births 1997–2017 and all cases were within 72 h. The proportion of extremely preterm infants (< 28 weeks gestation) rose steadily during the study period but there was no rise in infections due to Gram-negative organisms. The spectrum of cultured organisms changed after 72 h as commensal organisms started to emerge.

**Conclusion:**

There has been a decrease in the incidence of neonatal early-onset infections compared to previous studies in western Sweden. The incidence of GBS infections was not as low as in other reports. Further studies are needed to assess if screening-based intra partum antimicrobial prophylaxis instead of a risk factor-based approach for identifying candidates for intrapartum antimicrobial prophylaxis would be a better option for this study area.

**Key notes:**

- This study is one of the longest running follow-ups in the world, a follow-up of 43 years of early-onset neonatal infections.
- The incidence of early-onset GBS infections is higher in Western Sweden compared to other local reports.
- No difference in the incidence of early-onset GBS depending on the definition of early-onset being within 72 h or 7 days of life.

## Background

Early-onset (EO) neonatal infections is a major cause of mortality and morbidity [1]. Studies have shown an increase in EO infections due to Gram-negative bacteria, especially among very-low-birth-weight infants [2]. Group B Streptococcus (GBS) remains as one of the most common pathogen even though targeted interventions like intrapartum antimicrobial prophylaxis (IAP) have shown a significant reduction in the incidence of EO GBS infection [2–5]. A risk factor-based approach for identifying candidates for IAP was published in Sweden 2008 and is the current policy in Sweden [6]. Some cases, however do not present with any risk factors and in many other countries the strategy for IAP is based on prenatal screening of GBS in late pregnancy instead [3, 7, 8].

Early-onset (EO) infection has been defined in a variety of ways. Many international reports use the definition of EO infection being within 72 h after birth, whereas in studies on EO GBS the definition is often within the first week of life [2, 9–11].

Two studies on neonatal sepsis and meningitis – from the same region as the present study - have been performed. Documentation on neonatal invasive infection starting from 1975 makes our data one of the longest running databases on neonatal infections in the world. In the first study, including data from 1975 to 1986, the incidence of EO neonatal infection, within the first week of life, caused by recognized pathogens was 1.9/1000 live births (LB) with 17% case-fatality rate [12]. In the second study from 1987 to 1996, the incidence of EO infection had increased to 2.3/1000 LB but the case-fatality rate had declined to 9% [13]. One of the reasons for the increase in incidence was considered to be a better survival of prematurely born infants with a high susceptibility to infections and better survival due to improved neonatal care [13].

The aim of this study was to document the incidence and etiology of EO neonatal invasive infections and changes in mortality for the period 1997–2017 by making a comparison between the last 10 years (2008–2017) and 1997–2007 and to compare with two previous studies from the same population covering the period 1975–1996 [12, 13]. A second aim was to evaluate if a change in the definition on EO neonatal infections to < 72 h after birth, instead of within the first week of life, would lead to missing cases of EO GBS infections.

With follow-up of 43 years, from 1975 to 2017, we wanted to identify if targeted interventions and improved survival of very-low-birth-weight infants have had an impact on incidence, etiology and/or mortality.

## Methods

This was an epidemiological, retrospective study on EO neonatal invasive infections with the definition of EO being 0–6 days after birth. All infants, within a week after birth, from whom a pathogenic organism was isolated from blood or cerebrospinal fluid (CSF) during the years 1997–2017 were included in the study. At the time of birth their mothers had to be living in Gothenburg or five surrounding municipalities in western Sweden. The data was obtained from the Clinical microbiology laboratory at Sahlgrenska University Hospital, Gothenburg. This laboratory unit serves all the maternity wards and the pediatric units at the Sahlgrenska University Hospital, where parturients in Gothenburg and surrounding municipalities are delivered. Data on the patients´ characteristics including gestational age, birthweight, type of delivery, Apgar score, and the performance of a lumbar puncture (LP) as well as outcomes were collected from the patients medical records. Risk factors as maternal fever and premature ruptures of membranes were collected from the mothers´ medical records.

The total number of LB from the study area between 1997 and 2017 was 184,853 children, 95,089 male (51.4%). Neonatal mortality 0–6 days of age was 1.4/1000 LB. Population data was retrieved from “The Swedish Medical Birth Register” (www.socialsyrelsen.se) and Statistics Sweden (www.scb.se/en).

Our primary outcome was the etiology of EO neonatal sepsis and meningitis, as determined from blood and CSF cultures between 1997 and 2017. A comparison was made of the incidence of EO neonatal infections between the last 10 years (2008–2017) compared to 1997–2007. Regarding changes in the incidence due to GBS, *S. aureus* and aerobic Gram-negative rods, changes were assessed from 1975 – the first year from which data on neonatal infections in the study area were collected.

At the laboratory, culturing, isolating and identifying of bacteria and fungi was performed according to validated methods used in the clinical routine diagnostics.

Blood was drawn when central intravascular lines were put in place or drawn peripherally. As a routine a minimum of 1 ml blood should have been drawn for culture.

Organisms defined as recognized pathogen included the following Gram-positive aerobic or facultative anaerobic bacteria: *Staphylococcus aureus,* Group A, B and C streptococci, pneumococci, *Enterococcus* spp.*, Actinomyces* and *Listeria monocytogenes*, and the following Gram-negative aerobic or facultative anaerobic bacteria: *Escherichia coli, Klebsiella* spp.*, Haemophilus influenzae, Pseudomonas aeruginosa, Enterobacter* spp.*, Proteus mirabilis, Bacteroides* spp.*, Serratia marcescens* and fungi *(Candida* spp*).* The occurrence of these bacteria was in all cases regarded as the cause of infection.

Cultures with commensal species or species of less clear clinical significance like coagulase-negative staphylococci (CoNS), *Bacillus* spp. and *Burkholderia cepacia* had to meet both of the following criteria to be considered the cause of an infection: 1. At least two of the following signs and symptoms as a change from baseline: apnea, bradycardia (heart rate < 100/min), temperature < 36.5 or > 38 °C. 2. Appropriate antibiotics prescribed for > 120 h and a central intravascular line in place within 72 h before the culture was drawn.

Cultures identifying commensal bacteria or species of less clear clinical significance without fulfilling the above criteria were excluded from the study.

We chose not to follow the criteria stated by Centers for Disease Control that a minimum of two positive blood cultures are required for a commensal-related sepsis since two cultures are seldom drawn as a routine at our unit and the definition was changed in year 2008 [14].

Multiple positive cultures from a single infant yielding the same pathogen and the same antibiotic susceptibility profile were considered to represent the same infectious episode.

Our secondary outcome was mortality of neonatal infections. Death related to infection was defined as death occurring within 7 days of culturing and clinical signs and symptoms of infection being documented as the direct cause of death.

The study was approved by the Ethics Committee of Gothenburg.

### Statistical analysis

Demographic data were presented as mean or median after testing for normality with Kolmogorov-Smirnov test. Correlations between continuous outcomes were analysed using the Spearman test and Fisher’s exact test (two-tailed) was used for comparisons of proportions (http://www.socscistatistics.com/tests/fisher/Default2.aspx). Analyses were conducted in SPSS version 25.0 (IBM Corp). A *p* value < 0.05 was considered significant.

## Results

**Fig. 1:**
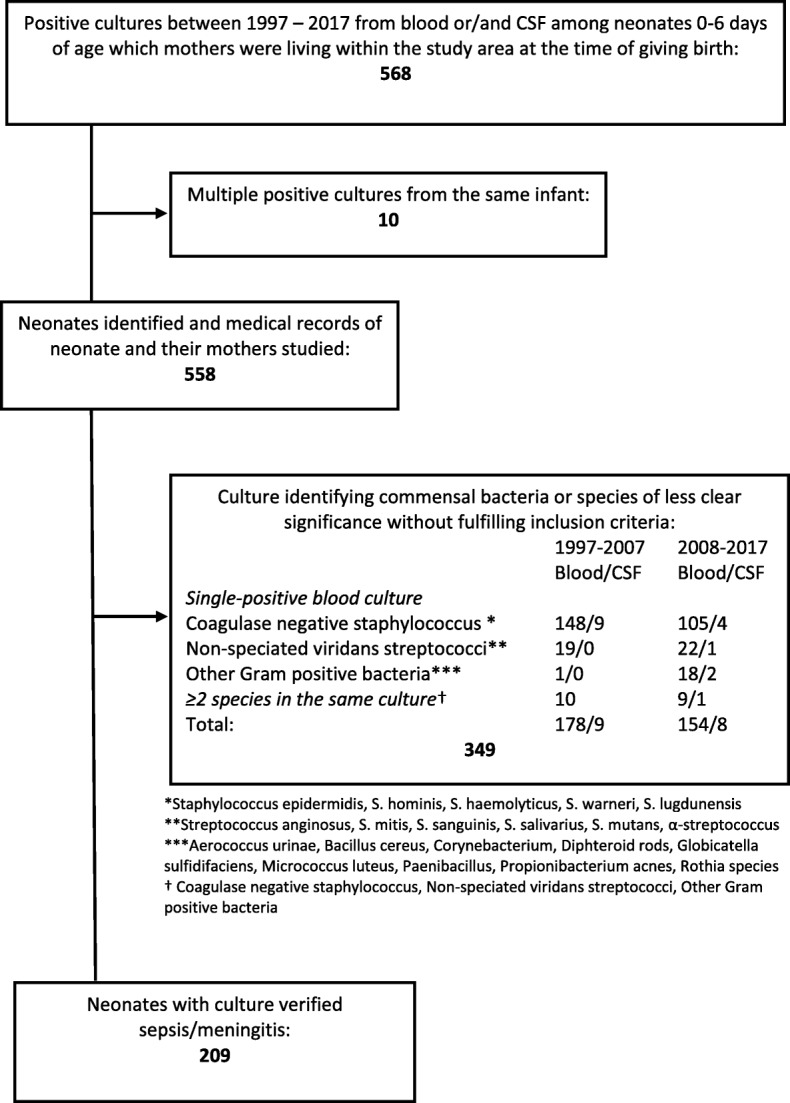
Flow chart over number of neonates included and excluded in the study

**Fig. 2:**
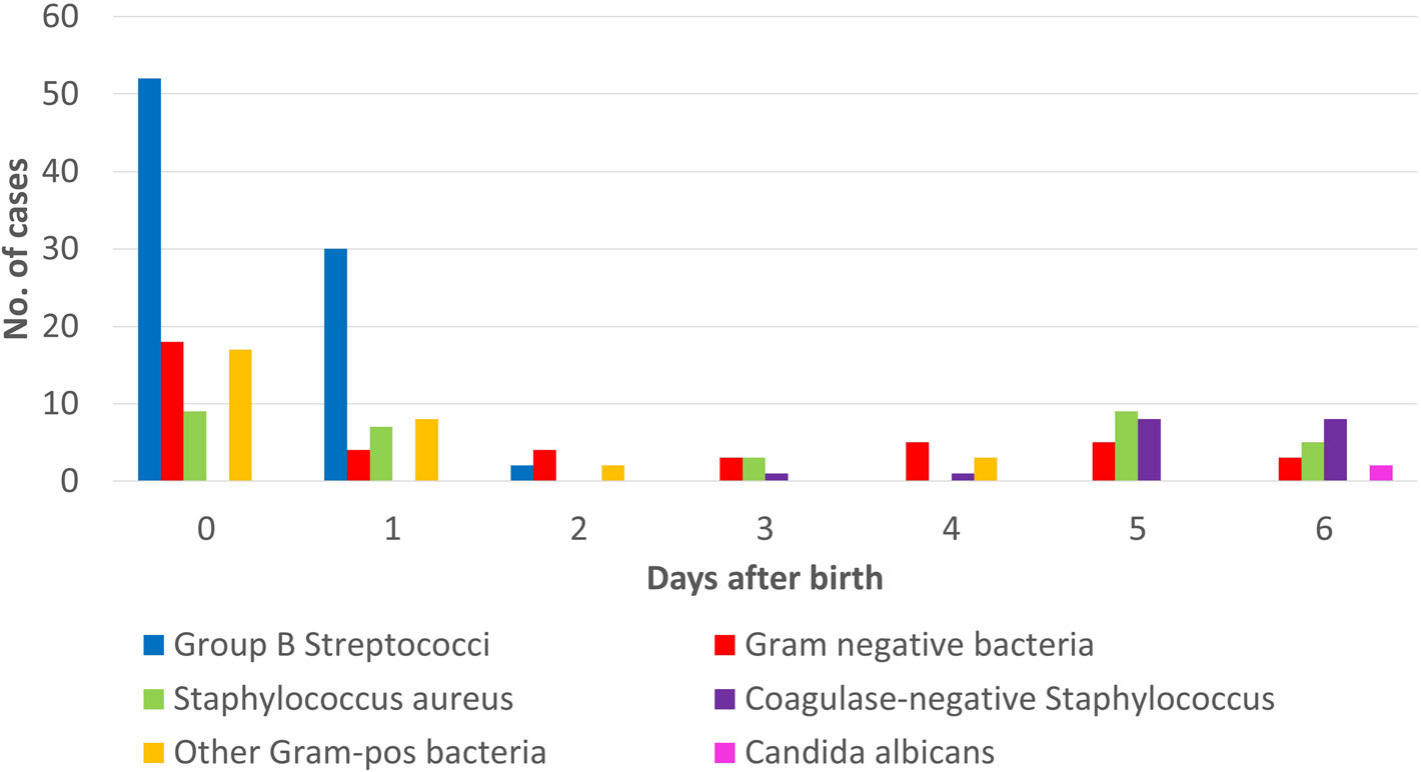
Age at onset of early-onset invasive infection according to organisms isolated from blood and/or cerebrospinal fluid among 209 neonates during the first 7 days of life between 1997 and 2017

Totally, 558 positive cultures were evaluated and 349 were excluded as contaminants, (Fig. **1**). During the period 1997–2017, there were 203 cases of EO sepsis and six cases of meningitis identified in 209 patients. The characteristics of infants according to gestational age are shown in Table 1. No case had missing information. The mean age of EO cases was 1.6 days (95%, CI 1.3–1.9). Most cases, or 160/209 (77%) occurred within 72 h, (Fig. **2****)**. The total incidence of EO infection, including both recognized pathogens and commensals as causative agents, was 1.1/1000 LB. The incidence declined from 1.4/1000 LB in 1997–2007 to 0.9/1000 LB in 2008–2017, (*p* = 0.004) and the case-fatality rate remained unchanged, (8/119 vs 7/90, (*p* = 0.8)) at 7%. The three most common organisms isolated from culture were GBS (40%), *S. aureus* (16%) and *E. coli* (9%), (Table **2**). The proportion of extremely preterm infants (< 28 weeks gestation) among the EO affected group rose steadily during the study period, and was significantly higher 2008–2017 compared to 1997–2007 (25/90 vs 12/119, *p* = 0.002).

**Table 1:** Characteristics of 209 infants with early-onset neonatal invasive infection between 1997 and 2017 according to gestational age. Comparison between period 1 (1997–2007) and period 2 (2008–2017)

		Gestational age (weeks)			
	Time period	< 28	28–36	≥ 37	Total
Birth weight (g) (Mean (CI))	1997–2017	781 (712–850)	1992 (1804–2180)	3638 (3533–3744)	2660 (2487–2832)
	Period 1 (1997–2007)	770 (644–896)	2101 (1850–2353)	3644 (3509–3778)	2874 (2663–3085)
	Period 2 (2008–2017)	786 (698–875)	1816 (1527–2104)	3629 (3453–3806)	2376 (2095–2658)
	*p*-value	1	0.1	0.3	0.004
Gender Male / Female n (%)	1997–2017	17 (46) / 20 (54)	33 (55) / 27 (45)	59 (53) / 53 (47)	109 (52) / 100 (48)
	Period 1 (1997–2007)	6 (50) / 6 (50)	21 (57) / 16 (43)	39 (56) / 31 (44)	66 (55) / 53 (45)
	Period 2 (2008–2017)	11 (44) / 14 (56)	12 (52) / 11 (48)	20 (48) / 22 (52)	43 (48) / 47 (52)
	*p*-value	1	0.8	0.4	0.3
PPROM^a^ n (%)	1997–2017	5 (14)	15 (25)	13 (12)	33 (16)
	Period 1 (1997–2007)	3 (25)	10 (27)	10 (14)	23 (19)
	Period 2 (2008–2017)	2 (8)	5 (22)	3 (7)	10 (11)
	*p*-value	0.3	0.8	0.4	0.1
Caeserean section n (%)	1997–2017	20 (54)	27 (45)	28 (25)	75 (36)
	Period 1 (1997–2007)	4 (33)	14 (38)	20 (29)	38 (32)
	Period 2 (2008–2017)	16 (64)	13 (57)	8 (19)	37 (41)
	*p*-value	0.2	0.2	0.4	0.2
Febrile mother n (%)	1997–2017	8 (22)	15 (25)	23 (21)	46 (22)
	Period 1 (1997–2007)	3 (25)	10 (27)	12 (17)	25 (21)
	Period 2 (2008–2017)	5 (20)	5 (22)	11 (26)	21 (23)
	*p*-value	1	0.8	0.3	0.7
Neonatal Distress Apgar < 7, n (%)					
at 5 min	1997–2017	15 (41)	11 (18)	10 (9)	36 (17)
	Period 1 (1997–2007)	3 (25)	7 (19)	6 (9)	16 (13)
	Period 2 (2008–2017)	12 (48)	4 (17)	4 (10)	20 (22)
	*p*-value	0.3	1	1	0.1
at 10 min	1997–2017	6 (16)	2 (3)	3 (3)	11 (5)
	Period 1 (1997–2007)	3 (25)	1 (3)	2 (3)	6 (5)
	Period 2 (2008–2017)	3 (12)	1 (4)	1 (2)	5 (6)
	*p*-value	0.4	1	1	1
Fatal cases n (%)	1997–2017	8 (22)	5 (8)	2 (2)	15 (7)
	Period 1 (1997–2007)	4 (33)	2 (5)	2 (3)	8 (7)
	Period 2 (2008–2017)	4 (16	3 (13)	0	7 (8)
	*p*-value	0.4	0.3	0.5	0.8
Total n (%)	1997–2017	37 (18)	60 (29)	112 (54)	209 (100)
	Period 1 (1997–2007)	12 (10)	37 (31)	70 (59)	119 (100)
	Period 2 (2008–2017)	25 (28)	23 (26)	42 (47)	90 (100)
	*p*-value	0.002	0.4	0.09	

^a^Premature rupture of membranes (> 18 h before birth)

**Table 2:** Organisms isolated from blood and/or cerebrospinal fluid from 209 neonates during the first 7 days of life between 1997 and 2017 according to gestational week

	Gestational age (weeks)			
Type of organism, n (%)	< 28	29–36	≥ 37	Total
Gram-pos bacteria:				
Group B Streptococci	9 (24.3)	23 (38.3)	52 (46.4)	84 (40.2)
*Staphylococcus aureus*	7 (18.9)	8 (13.3)	18 (16.1)	33 (15.8)
*Enterococcus* spp	0 (0)	0 (0)	12 (10.7)	12 (5.7)
Pneumococci	0 (0)	3 (5.0)	3 (2.7)	6 (2.9)
Coagulase-neg Staphylococci^a^	10 (27.0)	8 (13.3)	0 (0)	18 (8.6)
*Listeria monocytogenes*	1 (2.7)	3 (5.0)	0 (0)	4 (1.9)
Other Gram-pos bacteria^†^	0 (0)	1 (1.7)	2 (1.8)	3 (1.4)
Gram-neg bacteria:				
*Escherichia coli*	3 (8.1)	3 (5.0)	12 (10.7)	18 (8.6)
*Klebsiella pneumoniae*	3 (8.1)	1 (1.7)	2 (1.8)	6 (2.9)
*Klebsiella oxytoca*	0 (0)	2 (3.3)	0 (0)	2 (1.0)
*Haemophilus influenzae*	0 (0)	3 (5.0)	2 (1.8)	5 (2.4)
*Enterobacter* spp	0 (0)	0 (0)	2 (1.8)	2 (1.0)
*Serratia Marcescens*	2 (5.4)	1 (1.7)	0 (0)	3 (1.4)
*Pseudomonas* spp	0 (0)	2 (3.3)	4 (3.6)	6 (2.9)
Other Gram-neg. Bacteria^‡^	0 (0)	2 (3.3)	3 (2.7)	5 (2.4)
Fungi:				
*Candida albicans*	2 (5.4)	0 (0)	0 (0)	2 (1.0)
Total	37 (100)	60 (100)	112 (100)	209 (100)

^a^ including e.g. *S. epidermidis, S. hominis, S. capitis,* ^†^ Beta-hemolytic streptococci group A and C, *Actinomyces spp.*

^‡^ *Burkholdeira cepacia, Neisseria meningitidis, Bacteroides* spp.*, Proteus mirabilis* (fatal case)

**Fig. 3:**
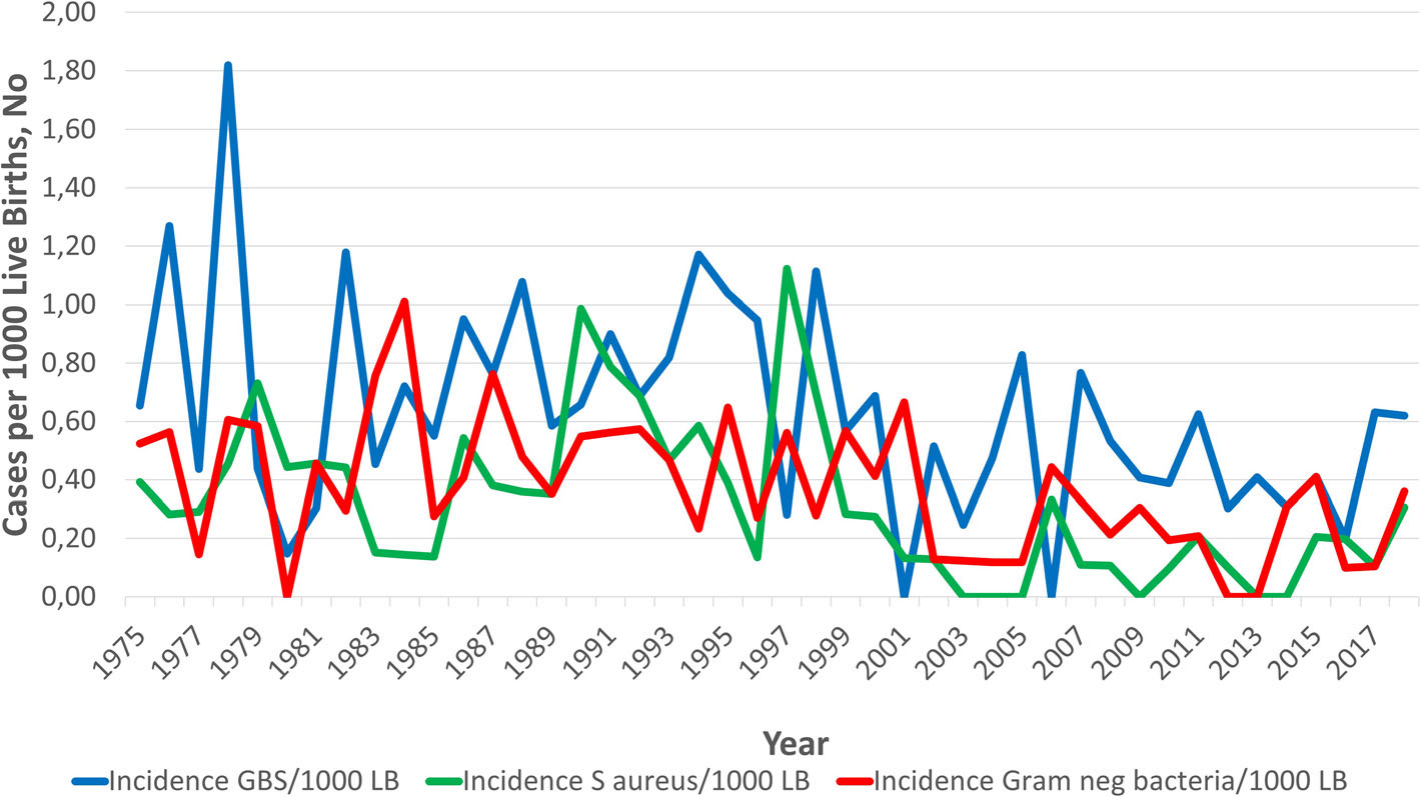
Changes in Incidence per 1000 live births of Early-Onset Invasive Infections caused by Group B Streptococcus, Staphylococcus aureus and aerobic Gram-negative rods between 1975 and 2017

During the years 1997–2017 the incidence of GBS, *S. aureus* and aerobic Gram negative rods was 154 cases (0.8 cases/1000 LB) compared to 295 cases (1.8 cases/1000 LB) 1975–1996 (*p* < 0.0001), (**Fig.** **3****)**.

Thirty-five (35/209, 17%) LPs yielded six cases with meningitis. One infant was born preterm in week 30 and had GBS cultured from CSF. The other five infants were all born at term. There were four cases of GBS, one case of *Enterococcus* and one case of *E. coli.* Mean age at diagnosis was 1.3 days. All infants, except the one with *Enterococcus* meningitis, had blood cultures yielding the same causative agent.

### Group B streptococci

The incidence of EO GBS infections 1997–2017 was 0.45/1000 LB and all cases of EO GBS were within 72 h, (Fig. **2****).** The majority, 52/84 (62%) of the infants were born at term. The mean birth weight was 3038 g (95% CI 2808–3269). The case-fatality rate was 8%, (7/84). Even though the incidence went from 0.5 cases/1000 LB 1997–2007 to 0.4 cases/1000 LB 2008–2017 the reduction in EO GBS infections after the publication of IAP guidelines in 2008 was not significant (*p* = 0.4). The incidence did not change after 2011 (0.4 cases/1000 LB), when the approach was implemented systematically.

### Gram-negative bacteria

The incidence of all EO Gram-negative infections was 0.25/1000 LB and the case-fatality rate was 13% (6/47). The incidence did not change between 1997 and 2007 and 2008–2017 (0.3/1000 LB vs. 0.2/1000 LB (*p* = 0.2)). The incidence of EO infections caused by aerobic Gram-negative rods during 1997–2017 was 0.2/1000 LB and the case-fatality rate was 11% (4/37). The most common agent was *E. coli* with an incidence of 0.1/1000 LB. Mean age at time of culture was 2.2 days (95% CI 1.48–2.95) and the mean birth weight was 2497 g (95% CI 2041–2953). Among the infants were 17/37 (46%) born prematurely.

### *Staphylococcus aureus*

Among the infants with EO *S. aureus* infection 15/33 (45%) were preterm. The mean birth weight was 2484 g (95% CI 2037–2931) and the mean age at time of culture was 2.8 days (95% CI 1.9–3.6). The incidence of *S. aureus* EO infections was 0.2/1000 LB and the case-fatality rate was 3%. The incidence declined from 0.3/1000 LB 1997–2007 to 0.1/1000 LB 2008–2017 (*p* = 0.013).

### Coagulase negative staphylocci

The incidence of EO CoNS infections was 0.1/1000 LB and it remained almost the same between 1997 and 2007 and 2008–2017 (0.08/1000 LB vs. 0.1/1000LB (*p* = 0.6)). All infants (18/18) with EO CoNS infection were born prematurely and the mean birth weight was 843 g (95% CI 719–1008). Mean age at time of culture was 5.3 days (95% CI 4.9–5.7). All cases with EO CoNS had a central line or had had a central line removed within 72 h of culture taken.

### Other gram-positive bacteria

The mean birth weight among infants with EO infections due to other Gram-positive bacteria was 3205 g (95% CI 2798–3612) and all had a positive culture drawn within 48 h after birth. The incidence of EO *Enterococcus* infections declined from 0.11/1000 LB during 1997–2007 to 0.02/1000 LB 2008–2017, (*p* = 0.017) but the incidence of other bacteria within this group remained unchanged.

### Fatal cases

Among the fatal cases 13/15 (87%) were born prematurely and their mean birth weight was 1518 g (95% CI 939–2098) compared to a prematurity rate of 84/194 (43%) and a mean birth weight of 2748 g (95% CI 2573–2924) among the infants who survived, (*p* = 0.002). The pathogens with the highest mortality were Gram-negative bacteria, *Klebsiella pneumoniae* at 33% (2/6) and *E. coli* at 11% (2/18). The only case of *Proteus mirabilis* infection was fatal. Even though the proportion of extremely preterm infants (< 28 weeks gestation) rose from 10% (12/119) 1997–2007 to 28% (25/90) 2008–2017 (*p* = 0.002) the case-fatality rate remained unchanged, (8/119 vs 7/90, (*p* = 0.8)).

## Discussion

This study shows that there has been a reduction in the incidence of neonatal EO invasive infections, including both recognized pathogens and commensals as causative agents, in the Gothenburg area from 1.4/1000 LB in 1997–2007 to 0.9/1000 LB in 2008–2017, (*p* = 0.004). Furthermore comparison to previous study from the same area showed a reduction from 2.3 cases/1000 LB in 1987–1996 to 1.1 cases/1000 LB in 1997–2017 (*p* < 0.0001) [13]. There was no statistical change in the case-fatality rate of 7% in 1997–2017 compared to 9% in 1987–1996 [13]. However when comparing the present study to the study from 1975 to 1986, when the incidence was 1.9 cases/1000 LB, the case-fatality rate was much higher at 17%, (*p* = 0.007) [12]. The reason for the decreasing incidence is probably due to general advances in neonatal care but the overall neonatal mortality at age 0–6 days in western Sweden went from 1.7/1000 LB in 1997–2007 to 1.2/1000 LB in 2008–2017 (*p* = 0), see Additional file 1 [15].

Studies from England and the United States have shown similar incidence as in our study. In a multicenter study from England with the definition of EO ≤ 48 h of age the incidence of EO sepsis was 0.9/1000 LB [16] and reports from the United States estimated the incidence, with the definition < 72 h, between 0.77–1/1000 LB [2, 17, 18]. A retrospective cohort from Australia showed an incidence of culture-proven EO infections with the definition < 72 h being 0.69/1000 LB but CoNS and other possible contaminants were not included [19]. A study from Norway reported a lower incidence of culture-confirmed EO infection, 0.5/1000 LB. However, they excluded all cases with single blood cultures yielding possible contaminants and CSF cultures were not considered [20].

The proportional rise of extremely preterm infants (< 28 weeks gestation) from 10 to 28% was probably due to a combination of factors. The percentage of premature neonates among LB infants had increased and they are more susceptibility to infections compared to term infants [1, 15]. At the same time the incidence among infants born closer to term decreased which might have been as a result of IAP.

The majority of EO cases due to recognized EO pathogens were cultured within 72 h. In our study, all EO GBS cases were diagnosed within 72 h after birth, and the spectrum of cultured organisms changed thereafter and organisms like CoNS and *Candida albicans* emerged as common causes of infection, (**Fig.** **2**). Infections after 72 h are more likely hospital-acquired, due to vascular access or other NICU-procedures.

Reasons for the high contamination rate of 349/558 (63%) is probably multi-factorial. There is a routine at our clinic that blood should be drawn for culture whenever a central line is put in place, whether there is a suspected infection or not. The diagnostic yield of cultures are influenced by the volume taken and from selected patients with reasonable suspicion of bacterial infection and most cases with contamination did not have any clinical suspicion of infection. Phlebotomy for blood culture is carried out by registered nurses, not by dedicated phlebotomy teams and they are often drawn at the same time as other blood tests to to minimize number of punctures of veins. It is important to follow policies and routines regarding blood-culture techniques with correct usage of appropriate antisepsis to avoid accidental inoculation of environmental and skin bacteria.

Six cases of meningitis were identified. All cases but one had the same bacteria cultivated in blood as in CSF. However, a LP was only performed in 35/209 (17%) of cases with a positive blood culture. LP is not recommended in asymptomatic full-term neonates and it is not performed if the neonate has no clinical signs suggesting meningitis [21]. This might be a problem as signs and symptoms of meningitis are often subtle. Even though meningitis in EO infections is quite uncommon a LP should be performed in any infant whose clinical course or laboratory data strongly suggest bacterial infection since it is the only way to rule out meningitis. Confirming the diagnosis of meningitis with cultures can be challenging given that a LP is often delayed and antibiotic treatment may result in negative cultures. Analysis of CSF by PCR for detection of bacterial DNA may be more sensitive in these situations, but the results of such diagnostic tests were not considered in the present study.

Many obstetricians in the region knew about the risk factor-based IAP guidelines and were acting accordingly before the publication of the guidelines in 2008 even though the approach was not implemented systematically until 2011. It is therefore impossible to pinpoint a specific year when the effect of risk factor-based IAP started to have a declining effect on EO GBS infections. EO GBS infections have been declining and the incidence during 1997–2017 at 0.45 cases/1000 LB was significantly lower compared to the previous study, covering the years 1987–1996, with an incidence of 0.9 cases/1000 LB, (*p* < 0.0001), (**Fig.** **3****)**.

Even though the incidence of EO GBS continued to decline further to 0.4 cases/1000 LB after the systematically start of risk factor-based IAP after 2011, the reduction is not as low as in other reports. A national population-based Swedish cohort study of EO GBS infections during 2006–2011 reported a decrease from 0.40 to 0.30 cases/1000 LB [5]. There might be a regional difference in the adherence to the guidelines or missed opportunities for IAP and that needs to be studied further.

A study from the United States, where screening-based IAP was implemented in 2002, reported an incidence of EO GBS at 0.23 cases/1000 LB in 2015 [22]. Although the studies are not comparable, the lower incidence in the United States as compared to our study may indicate that a screening-based IAP could have a greater impact on EO GBS infection than a risk factor-based approach. A study from London, United Kingdom, reported a decrease in incidence of EO GBS infection from 0.99/1000 LB with risk factor-based IAP to 0.33/1000 LB with screening-based IAP and after returning back to risk factor-based IAP the incidence went up again [23, 24]. Missed opportunities for prevention occurs both for risk-based IAP and screening-based IAP [5, 22].

Studies have shown an increase in EO Gram-negative sepsis, especially infections caused by *E. coli* [2]. Since very-low-birth-weight infants are more likely to have invasive infection due to Gram-negative organisms and *E. coli* in particular, rather than Gram-positive organisms like GBS, the increasing rate could be a reflection of improved survival of very-low-birth-weight infants [25, 26]. In our study we did not see a rise in infections due to Gram-negative organisms even though the number of singleton live born infants in Sweden weighing < 1000 g increased from 0.22% (1975–1987) to 0.38% (2008–2016), (*p* < 0.0001), **(Fig.** **3****)** [15].

One reason for the declining trend observed for *S. aureus* and aerobic Gram-negative rods as well as GBS might be that IAP affects the outcome of blood cultures. These infants get clinical signs of sepsis but cultures remain negative. During the last decade there has been a lot more focus on hygiene within neonatal units with increased awareness and surveillance of compliance to routines even though they are targeted towards affecting late-onset infections. These factors may have contributed to the overall declining trend in EO infections.

The main strength of this study is the long follow-up time of 43 years. The study includes both patients within a neonatal unit and infants within a community. However, only infants living within Gothenburg and surrounding municipalities are included which effects the study’s generalizability. The study is retrospective and diagnostic/reporting bias is always a concern especially with this long follow-up time and in regards of separating true infections from commensal bacteria. However, since this study only includes EO-infections the risk for this of kind of bias affecting the true incidence is small. In the first two studies from 1975 to 1986 and 1987–1996, CoNS and other commensal bacteria are excluded which could mean that the reduction in incidence of traditional neonatal EO invasive infections compared to previous studies might be underestimated. Regarding culture techniques, the systems used for blood culturing have developed and improved over the years, and this might have increased the ability to detect certain fastidious, “difficult to culture” microorganisms. However, the vast majority of the episodes of early onset neonatal sepsis are caused by bacteria which are easily cultured, detected, and identified in the laboratory, and have been so for many decades, e.g. GBS, *S. aureus*, CoNS, *E. coli* and other aerobic gram-negative rods. Therefore we do not believe that changes in culture techniques have had any impact on the incidence. Also, if there had been an impact of culture improvements, that would have resulted in an increased incidence over time, but we see the opposite pattern.

We included cases with a single blood-culture even though they showed commensal bacteria. This might have overestimated the incidence of infections caused by these bacteria. Low sample volume, especially in very-low-birth-weight infants, is a major caveat in clinical practice regarding blood cultures and decreases culture sensitivity. Since we only included culture-positive infections we might have underestimated the true incidence of neonatal infections.

## Conclusions

In conclusion the present study showed that the incidence of EO neonatal invasive infections has decreased compared to previous studies within the same population despite a proportional rise of extremely preterm infants (< 28 weeks gestation). The start of a risk factor-based approach for IAP may have contributed but the decrease in the incidence of EO GBS infections was not as significant as in other reports. Further studies are needed to assess if there are missed opportunities for IAP or if screening-based IAP would be a better option.

## Supplementary information

**Additional file 1.** List of live births within the study area along with the incidence of neonatal (0-6 days) mortality per 1000 live births per year within western Sweden between 1975 - 2017. Numbers were retrieved from the statistical database of The National Board of Health and Welfare, Stockholm, Sweden on 22-10-2019.

## Data Availability

The datasets generated and/or analysed during the current study are not publicly available due to that the ethics committee specifically state that no data, which can identify a patient can be publicly available but are available from the corresponding author on reasonable request.

## Abbreviations

CoNS: Coagulase-negative staphylococci
CSF: Cerebrospinal fluid
EO: Early onset
GBS: Group B streptococci
IAP: Intra partum antimicrobial prophylaxis
LB: Live births
LP: Lumbar puncture
