# Supplementary material for: Changes in incidence and etiology of early-onset neonatal infections 1997–2017 – a retrospective cohort study in western Sweden
Source: BMC Pediatr. 2019 Dec 12;19:490. doi: 10.1186/s12887-019-1866-z (PMC6907277; doi:10.1186/s12887-019-1866-z)
Supplement: Supplementary file 1 — Additional file 1. List of live births within the study area along with the incidence of neonatal (0-6 days) mortality per 1000 live births per year within western Sweden between 1975 - 2017. Numbers were retrieved from the statistical database of The National Board of Health and Welfare, Stockholm, Sweden on 22-10-2019. [file 12887_2019_1866_MOESM1_ESM.pdf]

APPENDIX - List of live births within the study area along with the incidence of neonatal (0-6 days) mortality per 1000 live births per year within western Sweden between 1975 - 2017. Numbers were retrieved from the statistical database of The National Board of Health and Welfare, Stockholm, Sweden on 22-10-2019.

Year	Male	Female	Total live births	Neonatal mortality (0-6 days)/1000 LB
1975	3914	3724	7638	4.8
1976	3559	3534	7093	5
1977	3629	3245	6874	5.2
1978	3321	3276	6597	4.1
1979	3502	3339	6841	4.5
1980	3460	3304	6764	3.3
1981	3363	3206	6569	3.1
1982	3433	3355	6788	3.3
1983	3447	3158	6605	3.2
1984	3552	3380	6932	2.6
1985	3751	3508	7259	3.3
1986	3838	3523	7361	2.9
1987	4029	3839	7868	3.3
1988	4354	3995	8349	2.9
1989	4376	4159	8535	2.6
1990	4659	4456	9115	3.4
1991	4534	4358	8892	2.7
1992	4619	4100	8719	3
1993	4328	4222	8550	2.3
1994	4328	4211	8539	2.5
1995	3938	3771	7709	2.3
1996	3851	3538	7389	2.2
1997	3708	3423	7131	2.4
1998	3692	3495	7187	1.5
1999	3661	3390	7051	2.1
2000	3765	3512	7277	1.2
2001	3921	3582	7503	1.9
2002	3912	3848	7760	1.4
2003	4182	3909	8091	1.3
2004	4420	3996	8416	2.4
2005	4391	4063	8454	1.3
2006	4587	4427	9014	1.6
2007	4665	4468	9133	1.2
2008	4815	4566	9381	1
2009	5029	4778	9807	1.3
2010	5274	5022	10296	1.2
2011	4932	4673	9605	0.9
2012	5062	4847	9909	1.1
2013	4915	4856	9771	1.7
2014	5078	4662	9740	1.5
2015	5019	4727	9746	1.1
2016	5137	4945	10082	1.3
2017	4924	4575	9499	1
Total	180874	170965	351839	
